# Transcriptional profiling of long non-coding RNAs regulating fruit cracking in *Punica granatum* L. under bagging

**DOI:** 10.3389/fpls.2022.943547

**Published:** 2022-10-11

**Authors:** Yuying Wang, Yujie Zhao, Yaqiong Wu, Xueqing Zhao, Zhaoxiang Hao, Hua Luo, Zhaohe Yuan

**Affiliations:** ^1^ Co-Innovation Center for Sustainable Forestry in Southern China, College of Forestry, Nanjing Forestry University, Nanjing, China; ^2^ Institute of Botany, Jiangsu Province and Chinese Academy of Sciences, Nanjing, China; ^3^ Zaozhuang Pomegranate Research Center, Institute of Botany, Zaozhuang, China

**Keywords:** pomegranate, bagging, lncRNA, target genes, functional analysis, fruit cracking

## Abstract

Fruit cracking tremendously damages the appearance of fruit, easily leads to pathogen invasion, greatly reduces the marketability and causes immense economic losses. The pivotal role of long non-coding RNAs (lncRNAs) in diverse biological processes has been confirmed, while the roles of lncRNAs underlying fruit cracking remain poorly understood. In this study, the incidence of fruit cracking was 7.26% under the bagging treatment, the control group was 38.11%, indicating that bagging considerably diminished the fruit cracking rate. LncRNA libraries for fruit cracking (FC), fruit non-cracking (FNC) and fruit non-cracking under bagging (FB) in pomegranate (*Punica granatum* L.) were performed and analysed *via* high-throughput transcriptome sequencing. A total of 3194 lncRNAs were obtained with a total length of 4898846 nt and an average length of 1533.77 nt in pomegranate. We identified 42 differentially expressed lncRNAs (DELs) and 137 differentially expressed mRNAs (DEGs) in FC vs FNC and 35 DELs and 160 DEGs in FB vs FC that formed co-expression networks respectively, suggesting that there are involved in phytohormone signaling pathway, lignin catabolic process, lipid transport/binding, cutin biosynthetic process and cell wall organization. We also found that 18 *cis*-acting DELs regulated 18 target genes, and 10 *trans*-acting DELs regulated 24 target genes in FC vs FNC, 23 DELs regulate 23 target genes for the cis-acting lncRNAs and 12 DELs regulated 36 target genes in FB vs FC, which provides an understanding for the regulation of the fruit cracking. Gene Ontology (GO) and Kyoto Encyclopedia of Genes and Genomes (KEGG) analysis results demonstrated that DELs participated in calcium ion binding, glycerophospholipid metabolism, flavonoid biosynthetic process, cell wall biogenesis, xyloglucan metabolic process, hormone signal transduction and starch and sucrose metabolism. Our findings provide new insights into the roles of lncRNAs in regulating the fruit cracking and lay the foundation for further improvement of pomegranate quality.

## Introduction

Pomegranate (*Punica granatum* L.) is a deciduous shrub or small tree of genus *Punica* in the Lythraceae family (Yuan et al., 2017; Lyu et al., 2020) and it is native to Central Asia such as Iran, Afghanistan and the Caucasus (Qin et al., 2017). Pomegranate possesses an outstanding ornamental and medicinal value and has become an emerging functional fruit due to the richness of ellagitannin-based compounds in the peel and aril, which have antioxidant, antibacterial, anti-inflammatory, anticancer, antidiabetic and cardiovascular health promoting properties (Vlachojannis et al., 2015; Baghel et al., 2021). At present, the research on pomegranate mainly focuses on evolution, ovule, flower, seed coat, fruit development (Saminathan et al., 2016; Yuan et al., 2017; Zhao et al., 2020; Qin et al., 2020), natural product biosynthesis (Ono et al., 2011), pericarp color synthesis (Zhao et al., 2015) and salt stress response (Liu et al., 2020). Few studies have focused on the quality of pomegranate peel appearance and its regulation mechanism.

Fruit cracking affects its appearance and quality, resulting in a decrease in the commercial value and marketability, resulting in economic loss (Ginzberg and Stern, 2019). Fruit cracking is an intricate biological process involving phytohormone signaling pathway, lipid metabolism, cutin biosynthetic process and cell wall organization, etc (Chen et al., 2019; Wang et al., 2019; Jiang et al., 2019). The outer layer of pericarp is covered with cutin and wax, which plays a vital role in resisting pathogens, reducing water loss and enhancing the storage and quality of fruit (Lewandowska et al., 2020; Li et al., 2021; Wang et al., 2022). Previous studies commonly reported to be prone to cracking include pomegranate (Galindo et al., 2014), cherry (Quero-García et al., 2021), grape (Zhang et al., 2021), apple (Joshi et al., 2018), jujube (Li et al., 2020b), Akebia trifoliate (Niu et al., 2020) and tomato (Capel et al., 2017). Fruit cracking is influenced by genetic factors (fruit genotype, cuticle and epidermal cells, cell wall, etc.) (Cortés et al., 1983; Correia et al., 2020), environmental factors (humidity, light and temperature, etc.) (Wang and Camp, 2000; Galindo et al., 2014), cultivation management practices (irrigating, bagging and spraying of plant growth regulators and minerals, etc.) (Joshi et al., 2018; Xue et al., 2020; Li et al., 2020c; Correia et al., 2020) and postharvest storage factors (hydrocooling and postharvest calcium treatment, etc.) (Wang and Long, 2015; Singh et al., 2021). The pomegranate fruit cracking was closely related to fruit volume and shape (Sharifani, 2014). Galindo et al. (Galindo et al., 2014) suggested that pomegranate fruits were sensitive to water deficit at the end of fruit growth and ripening period. Rainfall can greatly affect previously water-stressed pomegranate plants, and an increase in aril turgor stresses the pericarp and makes it susceptible to cracking. Joshi et al. (2021) showed that high growth rates weakened the pericarp and increased the susceptibility of the fruit to cracking. Multiple previous studies have shown that bagging immensely reduces the incidence of sunburn and fruit cracking (Griñán et al., 2019; Asrey et al., 2020). Hosein-Beigi et al. (2019) investigated the effect of calcium (Ca) (0, 0.75% and 1.5%), boron (B) (0 and 3000 ppm) and gibberellin (GA_3_) (0, 75 and 150 ppm) on ‘Malase-Torshe-Saveh’ pomegranate fruit, the application of boron can increase the water content of the peel, thereby combination spraying of Ca, B and GA_3_ was more effective than spraying alone, with the least symptoms of fruit cracking and sunburn.

Non-coding RNAs (ncRNAs) refers to the general term for RNAs that do not encode proteins, including ribosomal RNAs (rRNAs), transfer RNAs (tRNAs), microRNAs (miRNAs), long non-coding RNAs (lncRNAs), circular RNAs (circRNAs), etc (Kim and Sung, 2012). LncRNAs transcripts are longer than 200 nucleotides and no protein-encoding function (Ulitsky and Bartel, 2013; Laurent et al., 2015). LncRNAs were originally considered to be the ‘noise’ of genome transcription, without biological function (Ulitsky and Bartel, 2013; Fatica and Bozzoni, 2014; Chekanova, 2015). Recent studies have shown that lncRNAs can regulate gene expression at epigenetic, transcriptional and post-transcriptional levels, and are involved in a variety of crucial regulatory processes such as photomorphogenesis, fruit softing and ripening, which are closely related to the growth and development of plants (Hadjiargyrou and Delihas, 2013; Wang et al., 2014; Berry and Dean, 2015; Correa et al., 2018). With the speedy development of high-throughput sequencing, considerable number of lncRNAs have been widely identified in Arabidopsis thaliana (Liu et al., 2012; Zhu et al., 2014), Oryza sativa (Li et al., 2007), Solanum lycopersicum (Zhu et al., 2015; Xue et al., 2020), Medicago truncatula (Wang et al., 2015) and Ginkgo biloba (Wu et al., 2019b). However, the function of lncRNAs in fruit cracking is rarely limited. Thus, it is considerable and urgent to reveal and investigate the function of lncRNAs in fruit cracking of pomegranate.

The pomegranate cultivar used was *Punica granatum* L. cv. ‘Daqingpitian’, an excellent variety and main planted variety in Zaozhuang City, Shandong Province, accounting for about 80% of the total cultivation. The fruit of ‘Daqingpitian’ is large-sized, with bright pericarp and wonderful appearance, nevertheless, it is cracking-susceptible. Base on this, we performed high-throughput transcriptome sequencing to reveal lncRNAs involved in pomegranate fruit cracking, and carried out coexpression analysis of differentially expressed lncRNAs (DELs) and differentially expressed mRNAs (DEGs). Furthermore, the cis- and trans-target genes of DELs were explored to expound the putative biological functions. Those results provide a foundation for future research of the molecular mechanism of fruit cracking.

## Materials and methods

### Plant materials and bagging treatment

Pomegranate cultivars ‘Daqingpitian’ was used in our experiment. Pomegranate trees that were about 15 years old were selected from an orchard in Zaozhuang City, Shandong Province, China (34°77’ N, 117°56’ E). Fruits on ten trees were covered with white single-layer bags on 110 days after full bloom (DAFB), 2020, fruits on ten trees without bagging treatment. In addition, these trees were subject to the same orchard management strategies. Types and incidence of fruit cracking were investigated at the final fruit ripening stage. Total fruit numbers for bagging and control are 358 and 307, respectively. We collected samples of fruit from non-cracking (FNC) to fruit cracking (FC) and non-cracking under bagging (FB) during late ripening. Three non-cracking fruits from three pomegranate trees with the same direction of light were selected as a biological replicate, the cracking fruits, non-cracking fruits under bagging were the same as that of non-cracking fruits and three biological replicates of each group of samples. The pericarps were immediately isolated into 15 ml centrifuge tubes, snap frozen in liquid nitrogen for 10 min and stored at -80°C.

### RNA extraction and transcriptome sequencing

The total RNA from these pericarps (FNC, FC and FB) was extracted using a mirVana miRNA Isolation Kit (Ambion-1561) in accordance with the manufacturer’s instructions. The RNA integrity, and quality were checked using an Agilent 2100 Bioanalyzer (Agilent Technologies, Santa Clara, CA, USA). Following analysis was performed on samples with RNA integrity number (RIN)≥ 7. Then, 6 libraries were constructed and sequenced on an Illumina HiSeq™ X platform used for reference transcriptome sequencing. Trimmomatic software (Bolger et al., 2014) was used to filter out adapter, N and low-quality reads contained in raw reads to obtain clean reads. After comparing clean reads to the reference genome (ASM765513v2) using Hisat2 (Kim et al., 2019) and obtaining a bam file of the comparing results, the reads comparing to the genes were subsequently assembled using StringTie (Pertea et al., 2015) and the individually assembled transcripts from each sample were merged a complete transcript. The raw data was deposited in the National Center for Biotechnology Information (NCBI) Sequence Read Archive (http://www.ncbi.nlm.nih.gov/sra/) under accession number PRJNA773212.

### Identification of lncRNAs

The lncRNAs can be classified into intergenic lncRNA (lincRNA), intronic lncRNA, anti-sense lncRNA, sense-lncRNA, bidirectional-lncRNA and other types according to the position relationship between lncRNA and coding sequence. Based on the characteristics of lncRNAs, the candidate lncRNAs were obtained by a rigorous four-step screening method: (1) The cuffcompare software (Ghosh and Chan, 2016) was used to compare merged transcripts with reference transcripts one by one to identify the location types of the remaining transcripts. Then, through the screening of candidate lncRNA transcripts, the transcripts with ‘I’, ‘u’, ‘x’, and ‘o’ were reserved. (2) Transcripts were screened according to the length of more than 200 nt and the number of exons greater than or equal to 2. (3) The transcripts obtained were analyzed for coding ability prediction using four software, CPC2 (Kang et al., 2017), CNCI (Sun et al., 2013), Pfam (Sonnhammer et al., 1998) and PLEK (Li et al., 2014), and transcripts with coding potential were screened out. (4) For the species with known lncRNA, the lncRNA sequences predicted in the third step were aligned with the known lncRNA sequences by using BLASTN software. Quantitative analysis was performed after combining with known lncRNA sequences. For species with no known lncRNA, the lncRNA sequences obtained in the third step were directly used for quantitative analysis.

### Expression level quantification and DEG, DEL screening

The FPKM method (Roberts et al., 2011) eliminates the effect of transcript length and sequencing amount differences on the calculation of transcript expression, this method was applied to calculate the transcript expression level. Then, DESeq software (Anders and Huber, 2012) was used to standardize the number of counts of each sample mRNA, lncRNA and calculated the difference multiple, and negative binomial distribution test (NB) was used to test the difference significance of the reads number, and finally the difference mRNAs, lncRNAs were screened based on the difference multiple (|log2 FC| >1) and difference significance test results (P-value< 0.05).

### DELs and DEGs localization and coexpression network construction

A plot was generated to display the localization and abundance of DELs and DEGs in the pomegranate genome by Circos software (Krzywinski et al., 2009). Expression correlation was calculated based on DELs and DEGs expression data using Pearson correlation test, and pairs with correlation coefficients of not less than 0.8 and p values less than or equal to 0.05 were selected and considered that there was a co-expression relationship. Cytoscape (Shannon et al., 2003) was used to construct a co-expression network.

### Target gene prediction and enrichment analysis

Potential target genes of lncRNAs were predicted according to different regulatory patterns in cis or trans-acting, respectively. The principle of cis-target gene prediction is that the function of lncRNAs is related to their neighboring protein-coding genes. LncRNAs located upstream may intersect with promoters or other cis-acting elements of co-expressed genes, thus regulating gene expression at the transcriptional or post-transcriptional level; lncRNAs located in the 3’UTR or downstream of genes may be involved in other regulatory roles. All coding genes that were near the lncRNAs in the up- or down-stream 100 kb and significantly co-expressed with the lncRNA were defined as target genes. The function of trans-acting target genes for lncRNAs is not related to the location of the coding genes, but rather to the protein-coding genes they co-express. The target genes were predicted by the correlation analysis method between the expression of lncRNAs and protein-coding genes. To preferably comprehend functions of the lncRNA target genes, GO and KEGG enrichment analyses were carried out.

### Quantitative real-time PCR (qRT-PCR) validation

Ten DELs were randomly selected from the outcomes of the transcriptional research and qRT-PCR was performed on the Applied Biosystems 7500. Specific primers of ten DELs were designed (**Table S1**). The Actin of pomegranate (F: AGTCCTCTTCCAGCCATCTC and R: CACTGAGCACAATGTTTCCA) was used as the internal reference gene. All reactions were carried out in three biological replicates. The Amplification procedure of qRT-PCR was 95°C for 5 min; 95°C for 15 s, 58°C for 45 s, a total of 40 cycles. The relative expression level was normalized by 2^−ΔΔCT^ method (Livak and Schmittgen, 2001).

## Results

### Types and incidence of fruit cracking

**Figure 1:**
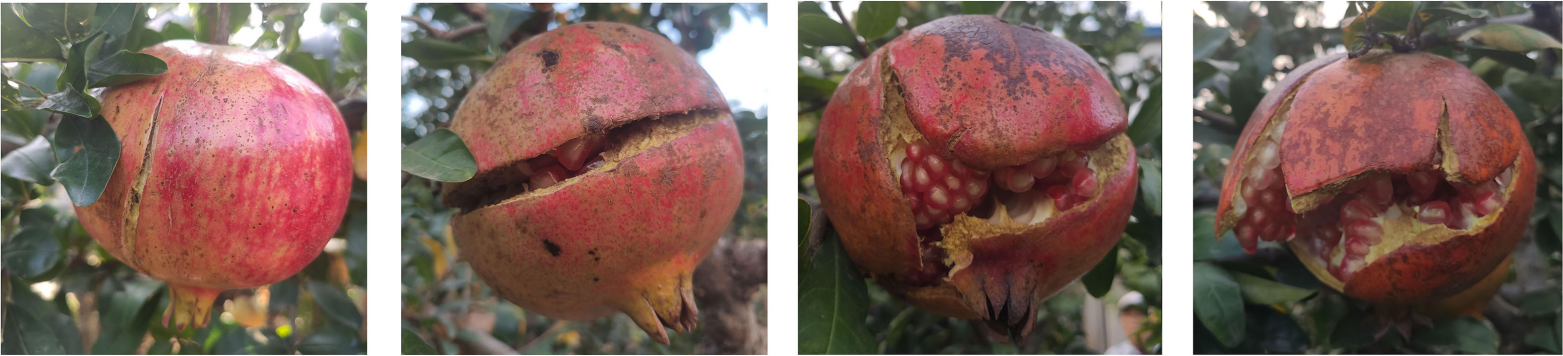
Types of fruit cracking.

The four types of fruit cracking in pomegranate was investigated, including longitudinal cracking, transversal cracking, longitudinal and transversal cracking and irregular cracking (**Figure 1**). Among them, the longitudinal cracking ratio was the highest about 70% (The number of longitudinal cracking fruits and total are 19 and 26 in fruit bagging, and 81 and 117 in control), and the other three were about 30%. We calculated that the incidence of fruit cracking under the bagging treatment was 7.26%, while the control group was as high as 38.11% (**Table 1**), indicating that bagging substantially reduced the fruit cracking rate.

**Table 1:** The incidence of fruit cracking under the bagging treatment and control.

Treatment	No.	Total	Number of cracked fruits	Fruit cracking rates
Fruit bagging	1	52	6	11.54%
	2	41	5	12.20%
	3	28	3	10.71%
	4	49	2	4.08%
	5	31	0	0
	6	36	0	0
	7	33	7	21.21%
	8	34	2	5.88%
	9	26	1	3.85%
	10	28	0	0
		358	26	7.26%
Control	1	51	28	54.90%
	2	18	5	27.78%
	3	21	10	47.62%
	4	14	1	7.14%
	5	24	10	41.67%
	6	21	2	9.52%
	7	25	11	44.00%
	8	29	18	62.07%
	9	48	20	41.67%
	10	56	12	21.43%
		307	117	38.11%

### RNA sequencing and identification of lncRNAs

**Figure 2:**
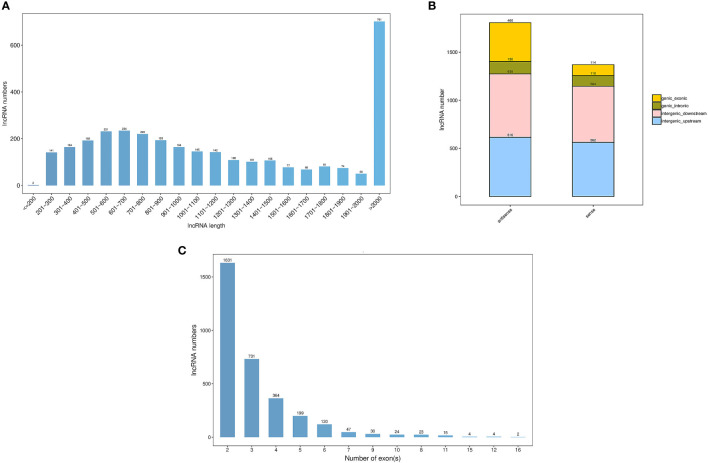
Summary of the characteristic long noncoding RNAs (lncRNAs) in pomegranate. **(A)** The length distribution of IncRNAs, **(B)** The classification of IncRNAs and **(C)** The exon number of IncRNAs.

In total, we obtained a total of 287.43 M, 282.83 M and 280.02 M raw reads from pomegranate fruit non-cracking under bagging (FB), fruit cracking (FC), fruit non-cracking (FNC), respectively (**Table 2**). After filtering out low quality sequences, approximately 284.96M, 280.5 M and 277.65 clean reads were generated from FB, FC and FNC, Q30 bases were distributed in 95.04~95.57%, with an average GC content of 50.08%. In addition, through coding potential calculator (CPC), coding-non-coding index (CNCI), protein families (Pfam) and predictor of long noncoding RNAs and messenger RNAs based on an improved k-mer scheme (PLEK) analysis, 3194 lncRNAs were identified with a total length of 4898846 nt and an average length of 1533.77 nt. The length distribution of lncRNAs showed that the maximum number of lncRNAs greater than 2000 bp was 701, accounting for 22% of the total number, and the minimum number of lncRNAs less than or equal to 200 bp was only 2 (**Figure 2A**). In addition, through the positional relationship between lncRNAs and known protein-coding transcripts, lncRNA types are counted from three levels, namely direction (sense and antisense), type (intergenic and genic), location (upstream, downstream, exonic and intronic). Among them, the direction was sense, and the number of lncRNAs in intergenic-upstream was at most 616 (**Figure 2B**). The exons number of lncRNAs indicated that the majority of lncRNAs contained 2 exons (**Figure 2C**).

**Table 2:** Quality of the sequencing data.

Sample	Raw Reads	Raw Bases	Clean Reads	Clean Bases	Valid Bases	Q30	GC
FB1	95.56M	14.33G	94.74M	13.01G	90.77%	95.41%	49.90%
FB2	93.34M	14.00G	92.53M	12.79G	91.37%	95.12%	49.64%
FB3	98.53M	14.78G	97.69M	13.55G	91.68%	95.04%	50.45%
FC1	95.65 M	14.35 G	94.85 M	12.98 G	90.44%	95.44%	50.07%
FC2	94.00 M	14.10 G	93.25 M	12.95 G	91.82%	95.44%	50.18%
FC3	93.18 M	13.98 G	92.40 M	12.79 G	91.53%	95.36%	50.22%
FNC1	96.12 M	14.42 G	95.32 M	13.15 G	91.18%	95.37%	50.28%
FNC2	92.18 M	13.83 G	91.42 M	12.70 G	91.82%	95.57%	50.12%
FNC3	91.72 M	13.76 G	90.91 M	12.65 G	91.93%	95.20%	49.85%

### LncRNAs expression level analysis

**Figure 3:**
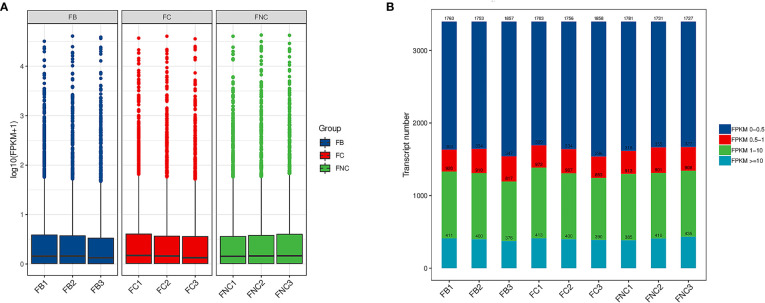
Long noncoding RNAs (lncRNAs) expression level analysis in pomegranate. **(A)** Boxplot of fragments per kilobase per million reads (FPKM) values. **(B)** Transcript expression b.

The fragments per kilobase per million reads (FPKM) was used to evaluate expression levels of 3194 lncRNAs transcripts in pomegranate FB, FC and FNC (**Table S2**). The overall expression levels of lncRNAs were different between FB, FC and FNC (**Figure 3A**). In addition, the results indicated differences in transcript numbers and distributions of gene expression values (**Figure 3B**). Approximately FPKM values of half of the transcripts were between 0 and 0.5 and about FPKM values of one-third of the transcripts were greater than or equal to 10.

### Differential expression and localization analysis

**Figure 4:**
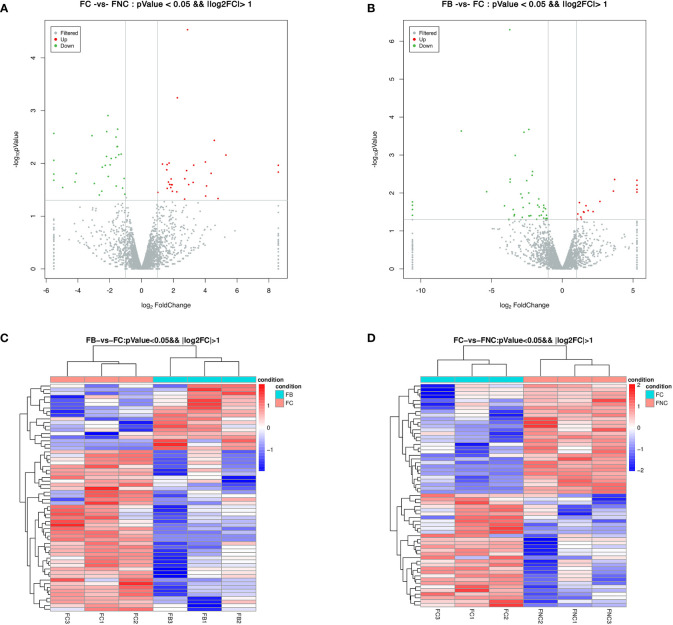
Differentially expressed lncRNAs (DELs) in pomegranate. Volcano plot of DELs in FC vs FNC **(A)** and FB vs FC **(B)**. Red and green dots represent significantly up-regulated and significantly down-regulated genes, respectively. Heat map of DELs based on hierarchical clustering analysis in FC vs FNC **(C)** and FB vs FC **(D)**. Red and blue indicate the highest and lowest expression level of DELs.

A total of 61 DELs were identified with a P-value< 0.05 and |log2 FC| > 1 in FC vs FNC (**Table S3**), 62 DELs in FB vs FC (**Table S4**). Among them, 31 were up-regulated and 30 were down-regulated in the FC vs FNC, 18 were up-regulated and 44 were down-regulated in the FB vs FC, as shown in a volcano plot (**Figures 4A, B**). Also, the expression patterns of the FC vs FNC, FB vs FC were compared, red and blue revealed the highest and lowest expression level, respectively. The heat map clustering showed that FC vs FNC, FB vs FC appear in the same cluster by hierarchical clustering analysis, indicating the genes in the same cluster may have similar biological functions (**Figures 4C, D**).

**Figure 5:**
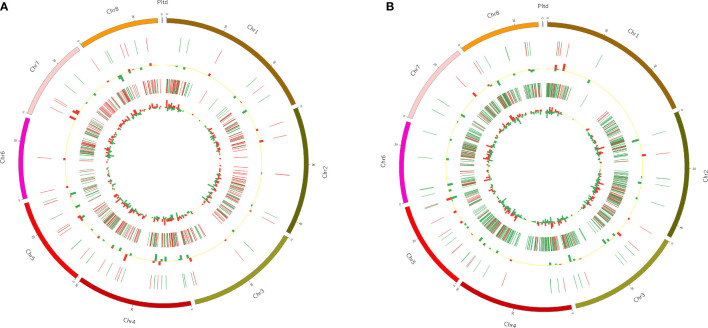
The distribution of differentially expressed lncRNAs (DELs) and differentially expressed mRNAs (DEGs) in FC vs FNC **(A)** and FB vs FC **(B)**. Among them, the outermost circle is the distribution of pomegranate chromosomes; the second circle is the distribution of DELs on chromosomes, the red line indicates up-regulation, and the green line indicates down-regulation; the third circle is a histogram of DELs at different positions, and red indicates up-regulation, green means down-regulation. The higher the column, the more the number of DELs; the fourth circle is the distribution of DEGs on chromosomes, the color distribution is the same as that of DELs; the innermost circle is the histogram of DEGs at different positions, the color distribution is same as DELs.

To display the position of DELs and DEGs more intuitively, Circos software was used to show them (**Figure 5**). The Circos diagram clearly revealed that the distribution of DELs and DEGs on chromosomes was not uniform. Among FC vs FNC, DELs were less distributed on chromosome 3 and chromosome 7, and were the least distributed on chromosome 6 (2), the same as FB vs FC; DELs were most distributed on chromosome 6 (12) (**Figure 5A**). In addition, the number of DELs distributed on chromosome 4 were the largest 12 in FB vs FC, followed by chromosome 5 (**Figure 5B**).

### Coexpression analysis of DELs and DEGs

**Figure 6:**
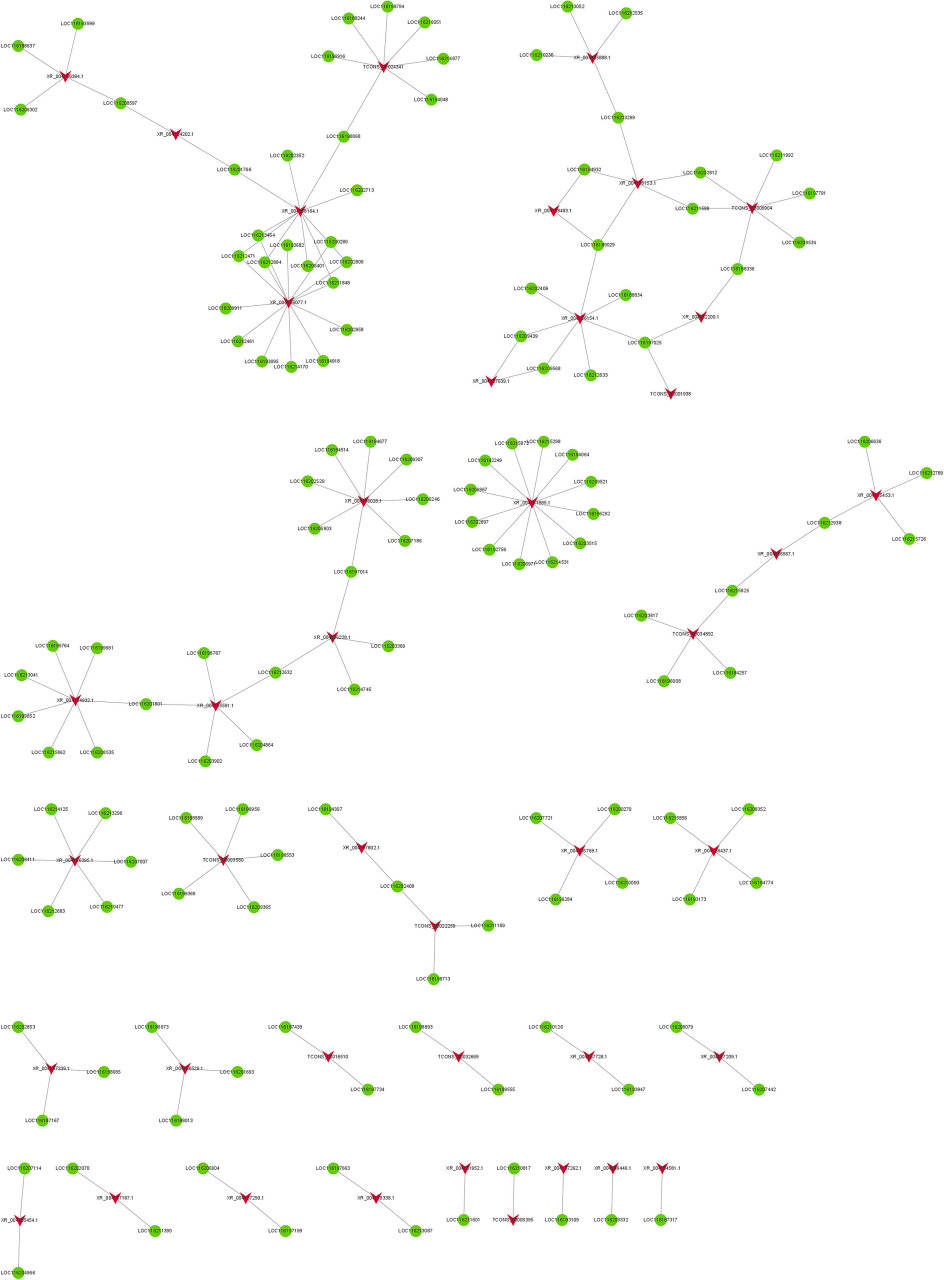
The co-expression network of differentially expressed lncRNAs (DELs) and differentially expressed mRNAs (DEGs) in FC vs FNC. The red arrow node and the green circular node represent DELs and DEGs.

To further explore the temporal and spatial transcription patterns and biological functions between DELs and DEGs, a coexpression network was constructed. The results suggested that a total of 42 DELs and 137 DEGs formed a coexpression network in FC vs FNC (**Figure 6**). Network analysis showed that one DEL co-regulated with one DEG or several DEGs and vice versa. It is suggested that there is a complex interaction between DELs and DEGs. Among them, XR_004158164.1 co-regulated with ten DEGs (LOC116201766, LOC116202352, LOC116198068, LOC116202713, LOC116213464, LOC116212471, LOC116212884, LOC116206401, LOC116211848 and LOC116200280) involved in ethylene-activated signaling pathway, lignin catabolic process, lipid transport/binding, cutin biosynthetic process and cell wall. XR_004156395.1 co-regulated with six DEGs (LOC116214125, LOC116208411, LOC116212683, LOC116210477, LOC116207007 and LOC116213296) involved in lipid transport/binding, cutin biosynthetic process and cell wall. TCONS_00001938 co-regulated with LOC116197025 (Polygalacturonase-1, PG1) involved in lipid transport, wax biosynthetic process and cell wall organization. In addition, XR_004157290.1 co-regulated with LOC116206904 (E3 ubiquitin-protein ligase-23, PUB23) and LOC116197199 (Polygalacturonase-2, PG2) involved in phytohormone signaling pathways, including ethylene-activated/abscisic acid- activated/jasmonic acid mediated signaling pathway.

**Figure 7:**
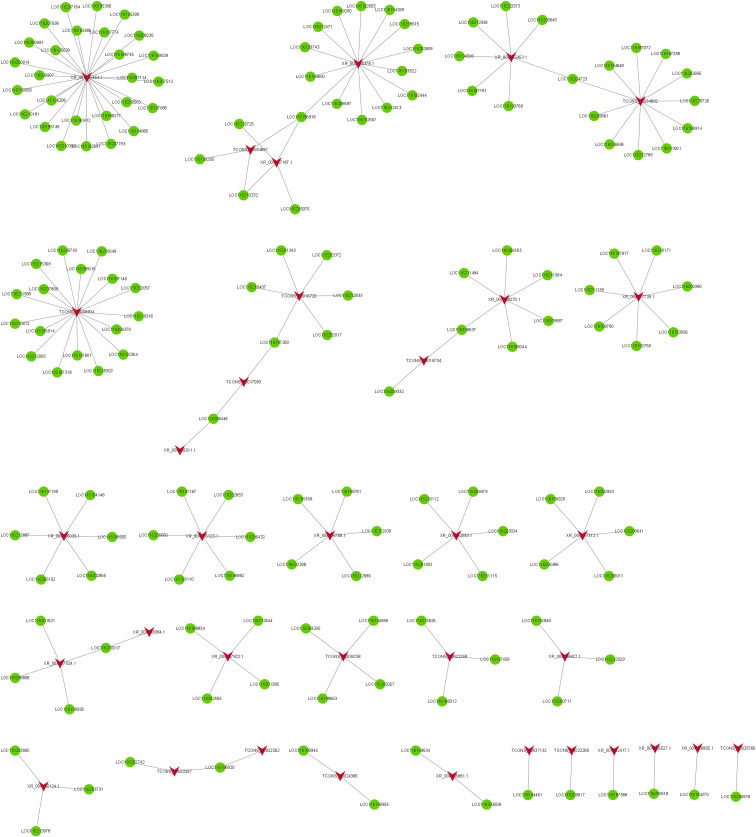
The co-expression network of differentially expressed lncRNAs (DELs) and differentially expressed mRNAs (DEGs) in FB vs FC. The red arrow node and the green circular node represent DELs and DEGs.

Among FB vs FC, 35 DELs and 160 DEGs constituted coexpression network (**Figure 7**). Among them, TCONS_00009904 co-regulated with 17 DEGs involved in lignin catabolic/biosynthetic process, secondary metabolic process, suberin biosynthetic process, cell wall biogenesis, flavonoid biosynthetic process, cell wall and xyloglucan:xyloglucosyl transferase activity. TCONS_00016729 co-regulated with six DEGs (LOC116187260, LOC116210407, LOC116201343, LOC116202072, LOC116212833 and LOC116202017) participate in secondary metabolic process, chitin catabolic process, cell wall macromolecule catabolic process, polysaccharide catabolic process, lignin catabolic/biosynthetic process, integral component of membrane and cell wall. In addition, TCONS_00017083, TCONS_00018960, TCONS_00022257 and XR_004156511.1 were all engaged in cell wall macromolecule catabolic process, polysaccharide catabolic process, lignin catabolic/biosynthetic process. XR_004155454.1, XR_004157024.1and XR_004157312.1 all engaged in flavonoid biosynthetic process, cell wall biogenesis, xyloglucan metabolic process and xyloglucan:xyloglucosyl transferase activity. As can be seen from the results of FC vs FNC and FB vs FC, phytohormone signaling pathways, lignin catabolic process, lipid transport/binding, cutin biosynthetic process and cell wall played significant roles in fruit non-cracking to fruit cracking. Fruit bagging affected flavonoid biosynthetic process, cell wall biogenesis, xyloglucan metabolic process and xyloglucan:xyloglucosyl transferase activity.

### Functional analysis of DEL target genes

**Figure 8:**
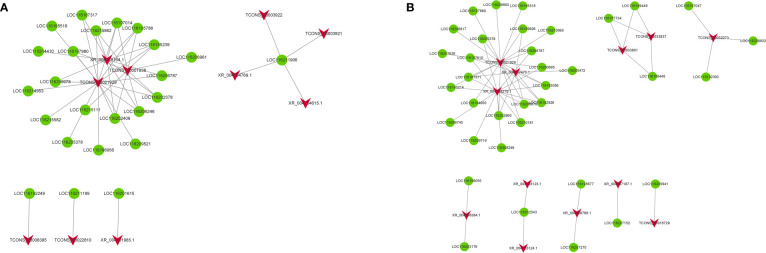
The coexpression network of trans-acting differentially expressed lncRNAs (DELs) and differentially expressed mRNAs (DEGs) in FC vs FNC **(A)** and FB vs FC **(B)**. The red arrow node and the green circular node represent DELs and DEGs.

Gene regulation may occur in the form of cis-/trans-acting. To explore the putative biological function of DELs, we divined the candidate targets of cis-/trans-acting lncRNA. For the cis-acting lncRNAs, 18 DELs regulate 18 target genes within the upstream and downstream 100 kb range in FC vs FNC (**Table S5**). For the trans-acting lncRNAs, the results revealed that there were 10 DELs regulated 24 target genes (**Table S6**; **Figure 8A**). In FB vs FC, 23 DELs regulate 23 target genes for the cis-acting lncRNAs (**Table S7**) and 12 DELs regulated 36 target genes (**Table S8** **;** **Figure 8B**). Among them, TCONS_00021929, XR_004158164.1 and TCONS_00001938 have complex regulatory relationships with 17, 1 and 10 target genes, respectively. Besides, four DELs (TCONS_00003922, TCONS_00003921, XR_004154769.1 and XR_004154615.1) have the same target gene LOC116211906. Three DELs (TCONS_00008395, TCONS_00022810, XR_004151985.1) displayed one-to-one associations with three DEGs (LOC116192249, LOC116211189, LOC116201615).

**Figure 9:**
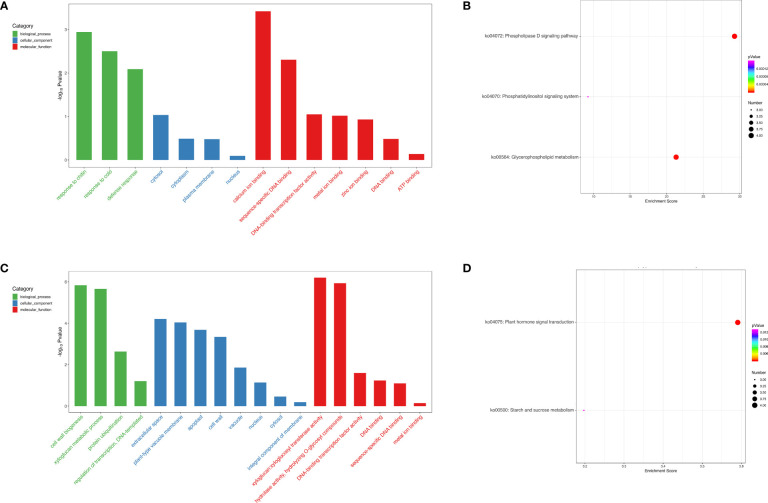
Functional Gene Ontology (GO) and Kyoto Encyclopedia of Genes and Genomes (KEGG) analysis of long non-coding RNA (lncRNA) target genes. GO enrichment analysis of lncRNA targets genes in FC vs FNC **(A)** and FB vs FC **(C)**. KEGG enrichment analysis of lncRNA targets genes FC vs FNC **(B)** and FB vs FC **(D)**.

To deeply investigate the function of the *cis*- and *trans*-targets of DELs, GO and KEGG pathways were carried out (**Figure 9**). GO analysis demonstrated that DELs participated in chitin, calcium ion binding, as well as metal ion binding in FC vs FNC (**Figure 9A**) and cell wall biogenesis, xyloglucan metabolic process and xyloglucan:xyloglucosyl transferase activity in FB vs FC (**Figure 9C**). KEGG analysis showed that DELs were involved in the phospholipase D signaling pathway (ko04072) and glycerophospholipid metabolism (ko00564) in FC vs FNC (**Figure 9B**) and plant hormone signal transduction (ko04075) and starch and sucrose metabolism (ko00500) in FB vs FC (**Figure 9D**).

### Quantitative real-time PCR (qRT-PCR) validation of DELs

To validate the reliability and authenticity of the RNA-seq data, ten DELs were randomly selected by quantitative real-time PCR (qRT-PCR) to determine their expression levels. The results showed that DELs displayed similar expression inclination to those obtained by the transcriptome data, which verify the reproducibility and reliability of transcriptomic analysis results (**Figure S1**). It would be helpful for further studies of the lncRNAs that involve in lipid transport, wax biosynthetic process, cell wall organization, phytohormone signaling pathways and calcium ion binding in fruit cracking and fruit non-cracking of pomegranate.

## Discussion

### Analysis of fruit cracking under bagging

Fruit cracking occurs during fruit development and greatly reduces the quality, shelf life and marketability of the fruit, resulting in tremendous economic losses. Among the factors that cause fruit cracking, in addition to environmental factors, the resistance of the fruit itself to cracking is crucial. Therefore, the mechanism of fruit cracking is still poorly understood.

There are multiple types of fruit cracking, such as longitudinal cracking, transversal cracking, longitudinal and transversal cracking, irregular cracking, calyx-end cracking, internal ring cracking, circular cracking and cuticular cracking (Ginzberg and Stern, 2019; Wang et al., 2021). In the present study, pomegranate fruit cracking types were longitudinal cracking, transversal cracking, longitudinal and transversal cracking, irregular cracking (**Figure 1**). Among them, the proportion of longitudinal cracking accounted for more than half of the amount. The fruit cracking rate of pomegranate under bagging treatment was less than 10%, while that of the control group was about 40%, indicating that bagging played a gigantic role in reducing the fruit cracking rate, which was consistent with previous studies (Griñán et al., 2019; Asrey et al., 2020).

### An dataset of lncRNAs associated with fruit cracking in pomegranate

LncRNAs exert an essential role in diverse biological processes such as leaf-color mutation, fruit development and ripening of plants and have been extensively studied in recent years (Zhang et al., 2018; Wu et al., 2019b). High-throughput sequencing technology for transcriptome analysis has been expeditiously ameliorated and dozens of lncRNAs have been identified in plants. For instance, 25,699, 3857, 6253 and 3679 lncRNAs were identified in Fragaria pentaphylla, Cucumis melo, Pyrus pyrifolia and Solanum lycopersicum, respectively (Zhu et al., 2015; Bai et al., 2019; Tian et al., 2019; Li et al., 2020a). Nevertheless, little is known about lncRNAs in pomegranate fruit cracking. In the present study, 3194 lncRNAs were identified with a total length of 4898846 nt and an average length of 1533.77 nt by strict filtration, among them, 61 DELs in FC vs FNC and 62 DELs in FB vs FC were differentially expressed. LncRNAs expression levels was confirmed by qRT-PCR analysis, which fully demonstrated the authenticity and accuracy of transcriptome results. Furthermore, we revealed that the distribution of DELs and DEGs on chromosomes was not uneven (**Figure 3**), this was consistent in the case of S. lycopersicum (Zhu et al., 2015).

### Functional analysis of DELs and DEGs

To further analyze the functions between DELs and DEGs, we constructed a coexpression network of 42 DELs and 137 DEGs in FC vs FNC (**Figure 6**) and 35 DELs and 160 DEGs in FB vs FC (**Figure 7**). The DEL XR_004158164.1 co-regulated with ten DEGs involved in ethylene-activated signaling pathway, lignin catabolic process, lipid transport/binding, cutin biosynthetic process and cell wall in FC vs FNC. The cuticle, composed of cutin and wax, is an essential part of the pericarp and plays a significant role in fruit cracking (Domínguez et al., 2011; Espaa et al., 2014; Petit et al., 2016; Jakobson et al., 2016). Ethylene is extensively involved in plant growth and development, and plays an essential regulatory role in fruit ripening, softening and cracking (Bapat et al., 2010; Chen et al., 2019). ERFs are located downstream of the ethylene signaling pathway, previous studies have shown that ERF4 was associated with fruit ripening and cracking in watermelon and tomato (Liao et al., 2019; Hu et al., 2022).

The main function of polygalacturonase (PG) is to hydrolyze polygalacturic acid, degrade pectin and disintegrate cell wall (Dautt-Castro et al., 2019). There is increasing evidence that PGs play an important role in fruit development in tomato, strawberry, apple, apricot and pear (Chen et al., 2016; Ke et al., 2018; Hou et al., 2019; Zhang et al., 2019; Paniagua et al., 2020). Dautt-Castro et al. (2019) found that MiPG14/17/21/22/49/69 were highly upregulated during fruit ripening. In addition, it was shown that PG was involved in pectin degradation in atemoya cv. African Pride, affecting fruit ripening and dehiscence (Chen et al., 2019). The PG homologs ARABIDOPSIS DEHISCENCE ZONE POLYGALACTURONASE1 (ADPG1) and ADPG2 were key factors causing fruit pod cracking in Arabidopsis thaliana (Ogawa et al., 2009). In our study, TCONS_00001938 co-regulated with LOC116197025 (Polygalacturonase-1, PG1) involved in lipid transport, wax biosynthetic process and cell wall organization. Zhu et al. (2020) found that genes related to cell wall metabolism and cuticle biosynthesis play a pivotal role in fruit cracking by performing peel transcriptome sequencing of the grape berry cv. Xiangfei (Vitis vinifera L.). In addition, XR_004157290.1 co-regulated with other genes such as LOC116197199 (Polygalacturonase-2, PG2) involved in ethylene-activated/abscisic acid-activated/jasmonic acid mediated signaling pathway to affect fruit cracking.

In FB vs FC, XR_004155454.1, XR_004157024.1and XR_004157312.1 were involved in flavonoid biosynthetic process, cell wall biogenesis, xyloglucan metabolic process and xyloglucan:xyloglucosyl transferase activity.

### *Cis*-/*trans*-acting of the lncRNAs and their target genes involved in lipid metabolism, chitin, and calcium ion binding, cell wall biogenesis, xyloglucan metabolic process

LncRNAs may regulate gene expression in the form of *cis-*/*trans*-acting manner (Fatica and Bozzoni, 2014). Cis-regulation refers to the transcriptional activation and expression regulation of adjacent mRNA by non-coding RNA, while trans, on the contrary, regulates the transcription of distal mRNA (Yan et al., 2017; Wu et al., 2019a). In this study, 18 DELs may regulate 18 target genes and 10 DELs regulated 24 target genes by cis-/trans-acting manner in FC vs FNC (**Figure 8A**), which participate in lipid metabolism and may be a crucial factor for fruit cracking. In addition, 23 DELs regulate 23 target genes for the cis-acting lncRNAs and 12 DELs regulated 36 target genes in FB vs FC (**Figure 8B**).

To investigate the function of the *cis*- and *trans*-targets of DELs, GO and KEGG pathways were carried out (**Figure 9**). GO analysis demonstrated that DELs participated in chitin, calcium ion binding, as well as metal ion binding in FC vs FNC and cell wall biogenesis, xyloglucan metabolic process and xyloglucan:xyloglucosyl transferase activity in FB vs FC. Calcium, an essential mineral element for plant growth and development, can interact with pectic acid to form calcium pectinate, which stabilizes the cell wall structure and strengthens the mechanical strength of the fruit pericarp, thus enhancing the fruit’s resistance to cracking (Hernández-Muñoz et al., 2006; Bakeer, 2016). Studies have shown that exogenous sprays of calcium or its compounds can reduce fruit cracking in pomegranates and cherries (Hosein-Beigi et al., 2019; Davarpanah et al., 2018). In addition, some metal ion (zinc and potash) also affect pomegranate cracking (Sharifani, 2014). KEGG analysis showed that DELs were involved in the phospholipase D signaling pathway (ko04072) and glycerophospholipid metabolism (ko00564). Phospholipase D hydrolyzes the phosphodiester bond of glycerol lipid phosphatidylcholine to produce phosphatidylic acid (Jenkins and Frohman, 2005). Glycospholipid metabolism belongs to lipid metabolism (Lu et al., 2022; Li et al., 2022), it suggests that lipid metabolism plays significant role in fruit cracking. In FB vs FC, DELs were participated in plant hormone signal transduction (ko04075) and starch and sucrose metabolism (ko00500), indicating bagging affected hormone biosynthesis, starch and sucrose metabolism, thereby influencing fruit dehiscence.

## Conclusion

In conclusion, the incidence of fruit cracking was 7.26% under the bagging treatment, the control group was as high as 38.11%, indicating that bagging substantially reduced the fruit cracking rate. In addition, a total of 3194 lncRNAs were obtained in pomegranate, 61 DELs and 62DELs were identified in FC vs FNC and FB vs FC. We constructed a coexpression network of 42 DELs and 137 DEGs in FC vs FNC and 35 DELs and 160 DEGs in FB vs FC, suggesting that there are involved in phytohormone signaling pathway, lignin catabolic process, lipid transport/binding, cutin biosynthetic process and cell wall organization. Moreover, the results demonstrated that 18 cis-acting DELs regulate 18 target genes, and 10 trans-acting DELs regulate 24 different target genes in FC vs FNC. Among FB vs FC, 23 DELs regulate 23 target genes for the cis-acting lncRNAs and 12 DELs regulated 36 target genes. GO and KEGG analysis results showed that DELs were involved in calcium ion binding, metal ion binding and glycerophospholipid metabolism in FC vs FNC, flavonoid biosynthetic process, cell wall biogenesis, xyloglucan metabolic process, hormone signal transduction and starch and sucrose metabolism in FB vs FC. This study provide insight into the molecular mechanism of fruit cracking in pomegranate, and lay a foundation for future research on fruit cracking of other fruits.

## Data availability statement

The original contributions presented in the study are publicly available. This data can be found here: NCBI, PRJNA773212.

## Author contributions

Conceptualization, YYW and ZY; Experimental operation, YYW and YZ; Writing—original draft preparation, YYW; Writing—review and editing YYW, YZ, YQW, XZ, ZH, and HL; Project administration, ZY. All authors have read and agreed to the published version of the manuscript.

## Funding

This work was supported by the Initiative Project for Talents of Nanjing Forestry University (GXL2014070, GXL2018032), the Priority Academic Program Development of Jiangsu High Education Institutions (PAPD), the Natural Science Foundation of Jiangsu Province (BK20180768), China Scholarship Council (CSC, 202108320304), Postgraduate Research & Practice Innovation Program of Jiangsu Province (KYCX22_1113).

## Conflict of interest

The authors declare that the research was conducted in the absence of any commercial or financial relationships that could be construed as a potential conflict of interest.

## Publisher’s note

All claims expressed in this article are solely those of the authors and do not necessarily represent those of their affiliated organizations, or those of the publisher, the editors and the reviewers. Any product that may be evaluated in this article, or claim that may be made by its manufacturer, is not guaranteed or endorsed by the publisher.

## Supplementary material

Supplementary Figure 1 The expression patterns of ten lncRNAs.

## References

[B1] Anders S. Huber W. (2012). Differential expression of RNA-seq data at the gene level-the DESeq package Vol. 10 (Heidelberg, Germany: European Molecular Biology Laboratory (EMBL), f1000research.

[B2] Asrey R. Kumar K. Sharma R. R. Meena N. K. (2020). Fruit bagging and bag color affects physico-chemical, nutraceutical quality and consumer acceptability of pomegranate (*Punica granatum* l.) arils. J. Food Sci. Tech. 57, 1469–1476. doi: 10.1007/s13197-019-04182-x PMC7054539 32180643

[B3] Baghel R. S. Keren-Keiserman A. Ginzberg I. (2021). Metabolic changes in pomegranate fruit skin following cold storage promote chilling injury of the peel. Sci. Rep. 11, 1–13. doi: 10.1038/s41598-021-88457-4 33911123 PMC8080622

[B4] Bai L. Chen Q. Jiang L. Lin Y. Ye Y. Liu P.. (2019). Comparative transcriptome analysis uncovers the regulatory functions of long noncoding RNAs in fruit development and color changes of *Fragaria pentaphylla* . Hortic. Res. 6, 15. doi: 10.1038/s41438-019-0128-4 30854215 PMC6397888

[B5] Bakeer S. M. (2016). Effect of ammonium nitrate fertilizer and calcium chloride foliar spray on fruit cracking and sunburn of manfalouty pomegranate trees. Sci. Hortic. 209, 300–308. doi: 10.1016/j.scienta.2016.06.043

[B6] Bapat V. A. Trivedi P. K. Ghosh A. Sane V. A. Ganapathi T. R. Nath P. (2010). Ripening of fleshy fruit: Molecular insight and the role of ethylene. Biotechnol. Adv. 28, 94. doi: 10.1016/j.scienta.2016.06.043 19850118

[B7] Berry S. Dean C. (2015). Environmental perception and epigenetic memory: mechanistic insight through FLC. Plant J. 83, 133–148. doi: 10.1111/tpj.12869 25929799 PMC4691321

[B8] Bolger A. M. Marc L. Bjoern U. (2014). Trimmomatic: a flexible trimmer for illumina sequence data. Bioinformatics 30, 2114–2120. doi: 10.1093/bioinformatics/btu170 24695404 PMC4103590

[B9] Capel C. Yuste-Lisbona F. J. López-Casado G. Angosto T. Cuartero J. Lozano R.. (2017). Multi-environment QTL mapping reveals genetic architecture of fruit cracking in a tomato RIL *Solanum lycopersicum* × *S. pimpinellifolium* population. Theor. Appl. Genet. 130, 213–222. doi: 10.1007/s00122-016-2809-9 27742924

[B10] Chekanova J. A. (2015). Long non-coding RNAs and their functions in plants. Curr. Opin. Plant Biol. 27, 207–216. doi: 10.1016/j.pbi.2015.08.003 26342908

[B11] Chen J. Duan Y. Hu Y. Li W. Xie J. (2019). Transcriptome analysis of atemoya pericarp elucidates the role of polysaccharide metabolism in fruit ripening and cracking after harvest. BMC Plant Biol. 19, 1–19. doi: 10.1186/s12870-019-1756-4 31132986 PMC6537181

[B12] Chen H. Shao H. Fan S. Ma J. Zhang D. Han M. (2016). Identification and phylogenetic analysis of the POLYGALACTURONASE gene family in apple. Hortic. Plant J. 2, 241–252. doi: 10.1016/j.hpj.2017.01.004

[B13] Correa J. P. O. Silva E. M. Nogueira F. T. S. (2018). Molecular control by non-coding RNAs during fruit development: from gynoecium patterning to fruit ripening. Front. Plant Sci. 9. doi: 10.3389/fpls.2018.01760 PMC6283909 30555499

[B14] Correia S. Santos M. Glińska S. Gapińska M. Matos M. Carnide V.. (2020). Effects of exogenous compound sprays on cherry cracking: Skin properties and gene expression. J. Sci. Food Agr. 100, 2911–2921. doi: 10.1002/jsfa.10318 32034777

[B15] Cortés C. Ayuso M. C. Palomares G. Cuartero J. Nuez F. (1983). Relationship between radial and concentric cracking of tomato fruit. Sci. Hortic. 21, 323–328. doi: 10.1016/0304-4238(83)90122-X

[B16] Dautt-Castro M. López-Virgen A. G. Ochoa-Leyva A. Contreras-Vergara C. A. Islas-Osuna M. A. (2019). Genome-wide identification of mango (*Mangifera indica* l.) polygalacturonases: Expression analysis of family members and total enzyme activity during fruit ripening. Front. Plant Sci. 10. doi: 10.3389/fpls.2019.00969 PMC6682704 31417586

[B17] Davarpanah S. Tehranifar A. Abadía J. Val J. Davarynejad G. Aran M.. (2018). Foliar calcium fertilization reduces fruit cracking in pomegranate (*Punica granatum* cv. ardestani). Sci. Hortic. 230, 86–91. doi: 10.1016/j.scienta.2017.11.023

[B18] Domínguez E. Cuartero J. Heredia A. (2011). An overview on plant cuticle biomechanics. Plant Sci. 181, 77–84. doi: 10.1016/j.plantsci.2011.04.016 21683870

[B19] Espaa L. Heredia-Guerrero J. Segado P. Benítez J. Heredia A. Domínguez E. (2014). Biomechanical properties of the tomato (*Solanum lycopersicum*) fruit cuticle during development are modulated by changes in the relative amounts of its components. New Phytol. 202, 790–802. doi: 10.1111/nph.12727 24571168

[B20] Fatica A. Bozzoni I. (2014). Long non-coding RNAs: new players in cell differentiation and development. Nat. Rev. Genet. 15, 7–21. doi: 10.1038/nrg3606 24296535

[B21] Galindo A. Rodríguez P. Collado-González J. Cruz Z. N. Torrecillas E. Ondoño S.. (2014). Rainfall intensifies fruit peel cracking in water stressed pomegranate trees. Agr. For. Meteorol. 194, 29–35. doi: 10.1016/j.agrformet.2014.03.015

[B22] Ghosh S. Chan C. K. K. (2016). Analysis of RNA-seq data using TopHat and cufflinks. Methods Mol. Biol. 1374, 339–361. doi: 10.1007/978-1-4939-3167-5_18 26519415

[B23] Ginzberg I. Stern R. A. (2019). Control of fruit cracking by shaping skin traits-apple as a model. Crit. Rev. Plant Sci. 38, 1–10. doi: 10.1080/07352689.2019.1698129

[B24] Griñán I. Morales D. Galindo A. Torrecillas A. Pérez-López D. Moriana A.. (2019). Effect of preharvest fruit bagging on fruit quality characteristics and incidence of fruit physiopathies in fully irrigated and water stressed pomegranate trees. J. Sci. Food Agr. 99, 1425–1433. doi: 10.1002/jsfa.9324 30129039 PMC6587789

[B25] Hadjiargyrou M. Delihas N. (2013). The intertwining of transposable elements and non-coding RNAs. Int. J. Mol. Sci. 14, 13307–13328. doi: 10.3390/ijms140713307 23803660 PMC3742188

[B26] Hernández-Muñoz P. Almenar E. Ocio M. J. Gavara. R. (2006). Effect of calcium dips and chitosan coatings on postharvest life of strawberries (*Fragaria* x *ananassa*). Postharv. Biol. Tec. 39, 247–253. doi: 10.1016/j.postharvbio.2005.11.006

[B27] Hosein-Beigi M. Zarei A. Rostaminia M. Erfani-Moghadam J. (2019). Positive effects of foliar application of Ca, b and GA_3_ on the qualitative and quantitative traits of pomegranate (*Punica granatum* l.) cv. 'Malase-Torshe-Saveh'. Sci. Hortic. 254, 40–47. doi: 10.1016/j.scienta.2019.04.081

[B28] Hou Y. Wu F. Zhao Y. Shi L. Zhu X. (2019). Cloning and expression analysis of polygalacturonase and pectin methylesterase genes during softening in apricot (*Prunus armeniaca* l.) fruit. Sci. Hortic. 256, 108607. doi: 10.1016/j.scienta.2019.108607

[B29] Hu Y. Sun H. Han Z. Wang S. Wang T. Li Q.. (2022). ERF4 affects fruit ripening by acting as a JAZ interactor between ethylene and jasmonic acid hormone signaling pathways. Hortic. Plant J. 8,1–11 doi: 10.1016/j.hpj.2022.01.002

[B30] Jakobson L. Lindgren L. O. Verdier G. Laanemets K. Brosché M. Beisson F.. (2016). BODYGUARD is required for the biosynthesis of cutin in arabidopsis. New Phytol. 211, 614–626. doi: 10.1111/nph.13924 26990896

[B31] Jenkins G. M. Frohman M. A. (2005). Phospholipase d: a lipid centric review. Cell. Mol. Life Sci. 62, 2305–2316. doi: 10.1007/s00018-005-5195-z 16143829 PMC11139095

[B32] Jiang F. Lopez A. Jeon S. De Freitas S. T. Yu Q. Wu Z.. (2019). Disassembly of the fruit cell wall by the ripening-associated polygalacturonase and expansin influences tomato cracking. Hortic. Res. 6, 17. doi: 10.1038/s41438-018-0105-3 30729007 PMC6355925

[B33] Joshi M. Baghel R. S. Fogelman E. Stern R. A. Ginzberg I. (2018). Identification of candidate genes mediating apple fruit-cracking resistance following the application of gibberellic acids 4 + 7 and the cytokinin 6-benzyladenine. Plant Physiol. Bioch. 127, 436–445. doi: 10.1016/j.plaphy.2018.04.015 29684828

[B34] Joshi M. Schmilovitch Z. Ginzberg I. (2021). Pomegranate fruit growth and skin characteristics in hot and dry climate. Front. Plant Sci. 12. doi: 10.3389/fpls.2021.725479 PMC8417319 34490023

[B35] Kang Y. J. Yang D. C. Kong L. Hou M. Meng Y. Q. Wei L.. (2017). CPC2: a fast and accurate coding potential calculator based on sequence intrinsic features. Nucleic Acids Res. 45, W12–W16. doi: 10.1093/nar/gkx428 28521017 PMC5793834

[B36] Ke X. Wang H. Li Y. Zhu B. Zang Y. He Y.. (2018). Genome-wide identification and analysis of polygalacturonase genes in *Solanum lycopersicum* . Int. J. Mol. Sci. 19 2290, 1–8. doi: 10.3390/ijms19082290 PMC6121401 30081560

[B37] Kim D. Paggi J. M. Park C. Bennett C. Salzberg S. L. (2019). Graph-based genome alignment and genotyping with HISAT2 and HISAT-genotype. Nat. Biotechnol. 37, 907–915. doi: 10.1038/s41587-019-0201-4 31375807 PMC7605509

[B38] Kim E. D. Sung S. (2012). Long noncoding RNA: unveiling hidden layer of gene regulatory networks. Trends Plant Sci. 17, 16–21. doi: 10.1016/j.tplants.2011.10.008 22104407

[B39] Krzywinski M. Schein J. Birol I. Connors J. Gascoyne R. Horsman D.. (2009). Circos: An information aesthetic for comparative genomics. Genome Res. 19, 1639–1645. doi: 10.1101/gr.092759.109 19541911 PMC2752132

[B40] Laurent G. S. Wahlestedt C. Kapranov P. (2015). The landscape of long noncoding RNA classification. Trends Genet. 31, 239–251. doi: 10.1016/j.tig.2015.03.007 25869999 PMC4417002

[B41] Lewandowska M. Keyl A. Feussner I. (2020). Wax biosynthesis in response to danger: its regulation upon abiotic and biotic stress. New Phytol. 227, 698–713. doi: 10.1111/nph.16571 32242934

[B42] Liao N. Hu Z. Li Y. Hao J. Zhang M. (2019). Ethylene-responsive factor 4 is associated with the desirable rind hardness trait conferring cracking resistance in fresh fruits of watermelon. Plant Biotechnol. J. 18, 1066–1077. doi: 10.1111/pbi.13276 31610078 PMC7061880

[B43] Li Q. Cheng C. Zhang X. Wang C. Yang S. (2020c). Preharvest bagging and postharvest calcium treatment affects superficial scald incidence and calcium nutrition during storage of ‘Chili’ pear (*Pyrus bretschneideri*) fruit. Postharv. Biol. Tec. 163, 111149. doi: 10.1016/j.postharvbio.2020.111149

[B44] Li N. Fu L. Song Y. Li J. Xue X. Li S.. (2020b). Wax composition and concentration in jujube (Ziziphus jujuba mill.) cultivars with differential resistance to fruit cracking. J. Plant Physiol. 255, 153294. doi: 10.1016/j.jplph.2020.153294 33070052

[B45] Li Z. Hu J. Wu Y. Wang J. Song H. Chai M.. (2022). Integrative analysis of the metabolome and transcriptome reveal the phosphate deficiency response pathways of alfalfa. Plant Physiol. Bioch. 170, 49–63. doi: 10.1016/j.plaphy.2021.11.039 34847401

[B46] Li L. Liu J. Liang Q. Zhang Y. Li Y. (2020a). Genome-wide analysis of long noncoding RNAs affecting floral bud dormancy in pears in response to cold stress. Tree Physiol. 41, 771–790. doi: 10.1093/treephys/tpaa147 33147633

[B47] Liu J. Jung C. Xu J. Wang H. Deng S. Bernad L.. (2012). Genome-wide analysis uncovers regulation of long intergenic noncoding RNAs in arabidopsis. Plant Cell 24, 4333–4345. doi: 10.1105/tpc.112.102855 23136377 PMC3531837

[B48] Liu C. Zhao Y. Zhao X. Dong J. Yuan Z. (2020). Genome-wide identification and expression analysis of the CLC gene family in pomegranate (*Punica granatum*) reveals its roles in salt resistance. BMC Plant Biol. 20, 560. doi: 10.21203/rs.3.rs-54027/v1 33308157 PMC7733266

[B49] Livak K. J. Schmittgen T. D. (2001). Analysis of relative gene expression data using real-time quantitative PCR and the 2(-delta delta C(T)) method. Methods 25, 402–408. doi: 10.1006/meth.2001.1262 11846609

[B50] Li L. Wang X. Rajkumar S. Viktor S. Wei D. Hang H.. (2007). Global identification and characterization of transcriptionally active regions in the rice genome. PLoS One 2, e294. doi: 10.1371/journal.pone.0000294 17372628 PMC1808428

[B51] Li F. Zhang X. Wang J. Jiang Y. Zhang X. Li X. (2021). Preharvest application of 1-methylcyclopropene and ethephon altered cuticular wax biosynthesis and fruit quality of apples at harvest and during cold storage. Hortic. Plant J. 8, 143–152. doi: 10.1016/j.hpj.2021.11.008

[B52] Li A. Zhang J. Zhou Z. (2014). PLEK: A tool for predicting long non-coding RNAs and messenger RNAs based on an improved k-mer scheme. BMC Bioinf. 15, 1–10. doi: 10.1186/1471-2105-15-311 PMC4177586 25239089

[B53] Lu M. Li Y. Jia H. Xi Z. Gao Q. Zhang Z. Z.. (2022). Integrated proteomics and transcriptome analysis reveal a decreased catechins metabolism in variegated tea leaves. Sci. Hortic. 295, 110824. doi: 10.1016/j.scienta.2021.110824

[B54] Lyu Y. Porat R. Yermiyahu U. Heler Y. Holland D. Dag A. (2020). Effects of nitrogen fertilization on pomegranate fruit, aril and juice quality. J. Sci. Food Agric. 100, 1678–1686. doi: 10.1002/jsfa.10182 31803940

[B55] Niu J. Shi Y. Huang K. Zhong Y. Chen J. Sun Z.. (2020). Integrative transcriptome and proteome analyses provide new insights into different stages of *Akebia trifoliata* fruit cracking during ripening. Biotechnol. Biofuels 13, 149. doi: 10.1186/s13068-020-01789-7 32843898 PMC7441727

[B56] Ogawa M. Kay P. Wilson S. Swain S. M. (2009). ARABIDOPSIS DEHISCENCE ZONE POLYGALACTURONASE1 (ADPG1), ADPG2, and QUARTET2 are polygalacturonases required for cell separation during reproductive development in arabidopsis. Plant Cell 21, 216–233. doi: 10.1105/tpc.108.063768 19168715 PMC2648098

[B57] Ono N. N. Britton M. T. Fass J. N. Nicolet C. M. Lin D. Tian L. (2011). Exploring the transcriptome landscape of pomegranate fruit peel for natural product biosynthetic gene and SSR marker discovery. J. Integr. Plant Biol. 53, 800–813. doi: 10.1111/j.1744-7909.2011.01073.x 21910825

[B58] Paniagua C. Ric-Varas P. García-Gago J. A. López-Casado G. Blanco-Portales R. Muñoz-Blanco J.. (2020). Elucidating the role of polygalacturonase genes in strawberry fruit softening. J. Exp. Bot. 71, 7103–7117. doi: 10.1093/jxb/eraa398 32856699

[B59] Pertea M. Pertea G. M. Antonescu C. M. Chang T. C. Mendell J. T. Salzberg S. L. (2015). StringTie enables improved reconstruction of a transcriptome from RNA-seq reads. Nat. Biotechnol. 33, 290–295. doi: 10.1038/nbt.3122 25690850 PMC4643835

[B60] Petit J. Bres C. Mauxion J. P. Fabienne W. J. T. Martin L. B. B. Fich E. A.. (2016). The glycerol-3-Phosphate acyltransferase GPAT6 from tomato plays a central role in fruit cutin biosynthesis. Plant Physiol. 171, 894–913. doi: 10.1104/pp.16.00409 27208295 PMC4902622

[B61] Qin G. Liu C. Li J. Qi Y. Gao Z. Zhang X.. (2020). Diversity of metabolite accumulation patterns in inner and outer seed coats of pomegranate: exploring their relationship with genetic mechanisms of seed coat development. Hortic. Res. 7, 10. doi: 10.1038/s41438-019-0233-4 31934341 PMC6946660

[B62] Qin G. Xu C. Ming R. Tang H. Guyot R. Kramer E. M.. (2017). The pomegranate (*Punica granatum* l.) genome and the genomics of punicalagin biosynthesis. Plant J. 91, 1108–1128. doi: 10.1111/tpj.13625 28654223

[B63] Quero-García J. Letourmy P. Campoy J. A. Branchereau C. Malchev S. Barreneche T.. (2021). Multi-year analyses on three populations reveal the first stable QTLs for tolerance to rain-induced fruit cracking in sweet cherry (*Prunus avium* l.). Hortic. Res. 8, 136. doi: 10.1038/s41438-021-00571-6 34059661 PMC8166915

[B64] Roberts A. Trapnell C. Donaghey J. Rinn J. L. Pachter L. (2011). Improving RNA-seq expression estimates by correcting for fragment bias. Genome Biol. 12, 1–14. doi: 10.1186/gb-2011-12-3-r22 PMC3129672 21410973

[B65] Saminathan T. Bodunrin A. Singh N. V. Devarajan R. Nimmakayala P. Jeff M.. (2016). Genome-wide identification of microRNAs in pomegranate (*Punica granatum* l.) by high-throughput sequencing. BMC Plant Biol. 16, 1–16. doi: 10.1186/s12870-016-0807-3 27230657 PMC4880961

[B66] Shannon P. Markiel A. Ozier O. Baliga N. S. Wang J. T. Ramage D.. (2003). Cytoscape: A software environment for integrated models of biomolecular interaction networks. Genome Res. 13, 2498–2504. doi: 10.1101/gr.1239303 14597658 PMC403769

[B67] Sharifani M. (2014). Description of biomechanical forces and physiological parameters of fruit cracking in pomegranate. Sci. Hortic. 178, 224. doi: 10.1016/j.scienta.2014.09.005

[B68] Singh V. Dan G. Parimi P. Kochanek B. Friedman H. (2021). Postharvest calcium treatment of apple fruit increased lenticel breakdown and altered cuticle structure. Postharv. Biol. Tec. 171, 111331. doi: 10.1016/j.postharvbio.2020.111331

[B69] Sonnhammer E. Eddy S. R. Ewan B. Alex B. Richard D. (1998). Pfam: Multiple sequence alignments and HMM-profiles of protein domains. Nucleic Acids Res. 26, 320–322. doi: 10.1093/nar/26.1.320 9399864 PMC147209

[B70] Sun L. Luo H. Bu D. Zhao G. Yu K. Zhang C.. (2013). Utilizing sequence intrinsic composition to classify protein-coding and long non-coding transcripts. Nucleic Acids Res. 41, e166. doi: 10.1093/nar/gkt646 23892401 PMC3783192

[B71] Tian Y. Bai S. Dang Z. Hao J. Zhang J. Hasi A. (2019). Genome-wide identification and characterization of long non-coding RNAs involved in fruit ripening and the climacteric in *Cucumis melo* . BMC Plant Biol. 19, 369. doi: 10.1186/s12870-019-1942-4 31438855 PMC6704668

[B72] Ulitsky I. Bartel D. P. (2013). lincRNAs: Genomics, evolution, and mechanisms. Cell 154, 26–46. doi: 10.1016/j.cell.2013.06.020 23827673 PMC3924787

[B73] Vlachojannis C. Erne P. Schoenenberger A. W. Chrubasik-Hausmann S. (2015). A critical evaluation of the clinical evidence for pomegranate preparations in the prevention and treatment of cardiovascular diseases. Phytother. Res. 29, 501–508. doi: 10.1002/ptr.5280 25611333

[B74] Wang S. Y. Camp M. J. (2000). Temperatures after bloom affect plant growth and fruit quality of strawberry. Sci. Hortic. 85, 183–199. doi: 10.1016/S0304-4238(99)00143-0

[B75] Wang Y. Fan X. Lin F. He G. Terzaghi W. Zhu D. (2014). Arabidopsis noncoding RNA mediates control of photomorphogenesis by red light. P. Nati. Acad. Sci. U.S.A. 111, 10359–10364. doi: 10.1073/pnas.1409457111 PMC4104870 24982146

[B76] Wang J. Gao X. Ma Z. Chen J. Liu Y. (2019). Analysis of the molecular basis of fruit cracking susceptibility in *Litchi chinensis* cv. baitangying by transcriptome and quantitative proteome profiling. J. Plant Physiol. 234, 106–116. doi: 10.1016/j.jplph.2019.01.014 30753966

[B77] Wang Y. Guo L. Zhao X. Zhao Y. Hao Z. Luo H.. (2021). Advances in mechanisms and omics pertaining to fruit cracking in horticultural plants. Agronomy 11, 1045. doi: 10.3390/agronomy11061045

[B78] Wang T. Z. Liu M. Zhao M. G. Chen R. Zhang W. H. (2015). Identification and characterization of long non-coding RNAs involved in osmotic and salt stress in *Medicago truncatula* using genome-wide high-throughput sequencing. BMC Plant Biol. 15, 131. doi: 10.1186/s12870-015-0530-5 26048392 PMC4457090

[B79] Wang Y. Long L. E. (2015). Physiological and biochemical changes relating to postharvest splitting of sweet cherries affected by calcium application in hydrocooling water. Food Chem. 181, 241–247. doi: 10.1016/j.foodchem.2015.02.100 25794746

[B80] Wang Y. Yang X. Chen Z. Zhang J. Si K. Xu R.. (2022). Function and transcriptional regulation of CsKCS20 in the elongation of very-long-chain fatty acids and wax biosynthesis in *Citrus sinensis* flavedo. Hortic. Res. 9, uhab027. doi: 10.1093/hr/uhab027 PMC8824539 35039844

[B81] Wu Y. Guo J. Wang T. Cao F. Wang G. (2019b). Transcriptional profiling of long noncoding RNAs associated with leaf-color mutation in *Ginkgo biloba* l. BMC Plant Biol. 19, 1–13. doi: 10.1186/s12870-019-2141-z 31783794 PMC6884798

[B82] Wu X. Shi T. Iqbal S. Zhang Y. Liu L. Gao Z. (2019a). Genome-wide discovery and characterization of flower development related long non-coding RNAs in *Prunus mume* . BMC Plant Biol. 19, 1–17. doi: 10.1186/s12870-019-1672-7 30744565 PMC6371585

[B83] Xue L. Sun M. Wu Z. Yu L. Yu Q. Tang Y.. (2020). LncRNA regulates tomato fruit cracking by coordinating gene expression *via* a hormone-redox-cell wall network. BMC Plant Biol. 20, 162. doi: 10.1186/s12870-020-02373-9 32293294 PMC7161180

[B84] Yan P. Luo S. Lu J. Y. Shen X. (2017). Cis- and trans-acting lncRNAs in pluripotency and reprogramming. Curr. Opin. Genet. Dev. 46, 170–178. doi: 10.1016/j.gde.2017.07.009 28843809

[B85] Yuan Z. Fang Y. Zhang T. Fei Z. Han F. Liu C.. (2017). The pomegranate (*Punica granatum* l.) genome provides insights into fruit quality and ovule developmental biology. Plant Biotechnol. J. 16, 1363–1374. doi: 10.1111/pbi.12875 PMC5999313 29271050

[B86] Zhang G. Chen D. Zhang T. Duan A. Zhang J. He C. (2018). Transcriptomic and functional analyses unveil the role of long non-coding RNAs in anthocyanin biosynthesis during sea buckthorn fruit ripening. DNA Res. 25, 465–476. doi: 10.1093/dnares/dsy017 29873696 PMC6191307

[B87] Zhang C. Cui L. Zhang P. Dong T. Fang J. (2021). Transcriptome and metabolite profiling reveal that spraying calcium fertilizer reduces grape berry cracking by modulating the flavonoid biosynthetic metabolic pathway. Food Chem.: Mol. Sci. 2, 100025. doi: 10.1016/j.fochms.2021.100025 PMC8991952 35415636

[B88] Zhang S. Ma M. Zhang H. Zhang S. Qian M. Zhang Z.. (2019). Genome-wide analysis of polygalacturonase gene family from pear genome and identification of the member involved in pear softening. BMC Plant Biol. 19, 1–12. doi: 10.1186/s12870-019-2168-1 31881836 PMC6935220

[B89] Zhao Y. Liu C. Ge D. Yan M. Yuan Z. (2020). Genome-wide identification and expression of YABBY genes family during flower development in *Punica granatum* l. Gene 752, 144784. doi: 10.1016/j.gene.2020.144784 32439372

[B90] Zhao X. Yuan Z. Feng L. Fang Y. (2015). Cloning and expression of anthocyanin biosynthetic genes in red and white pomegranate. J. Plant Res. 128, 687–696. doi: 10.1007/s10265-015-0717-8 25810223

[B91] Zhu Q.H. Stephen S. Taylor J. Helliwell C. A. Wang M. B. (2014). Long noncoding RNA s responsive to fusarium oxysporum infection in *Arabidopsis thaliana* . New Phytol. 201, 574–584. doi: 10.1111/nph.12537 24117540

[B92] Zhu B. Yang Y. Li R. Fu D. Wen L. Luo Y.. (2015). RNA Sequencing and functional analysis implicate the regulatory role of long non-coding RNAs in tomato fruit ripening. J. Exp. Bot. 66, 4483–4495. doi: 10.1093/jxb/erv203 25948705 PMC4507755

[B93] Zhu M. Yu J. Zhao M. Wang M. Yang G. (2020). Transcriptome analysis of metabolisms related to fruit cracking during ripening of a cracking-susceptible grape berry cv. xiangfei (*Vitis vinifera* l.). Genes Genom. 42, 639–650. doi: 10.1007/s13258-020-00930-y 32274647

