# Supplementary figures and images for: Transcriptional profiling of long non-coding RNAs regulating fruit cracking in Punica granatum L. under bagging
Source: Front Plant Sci. 2022 Oct 11;13:943547. doi: 10.3389/fpls.2022.943547 (PMC9592827; doi:10.3389/fpls.2022.943547)

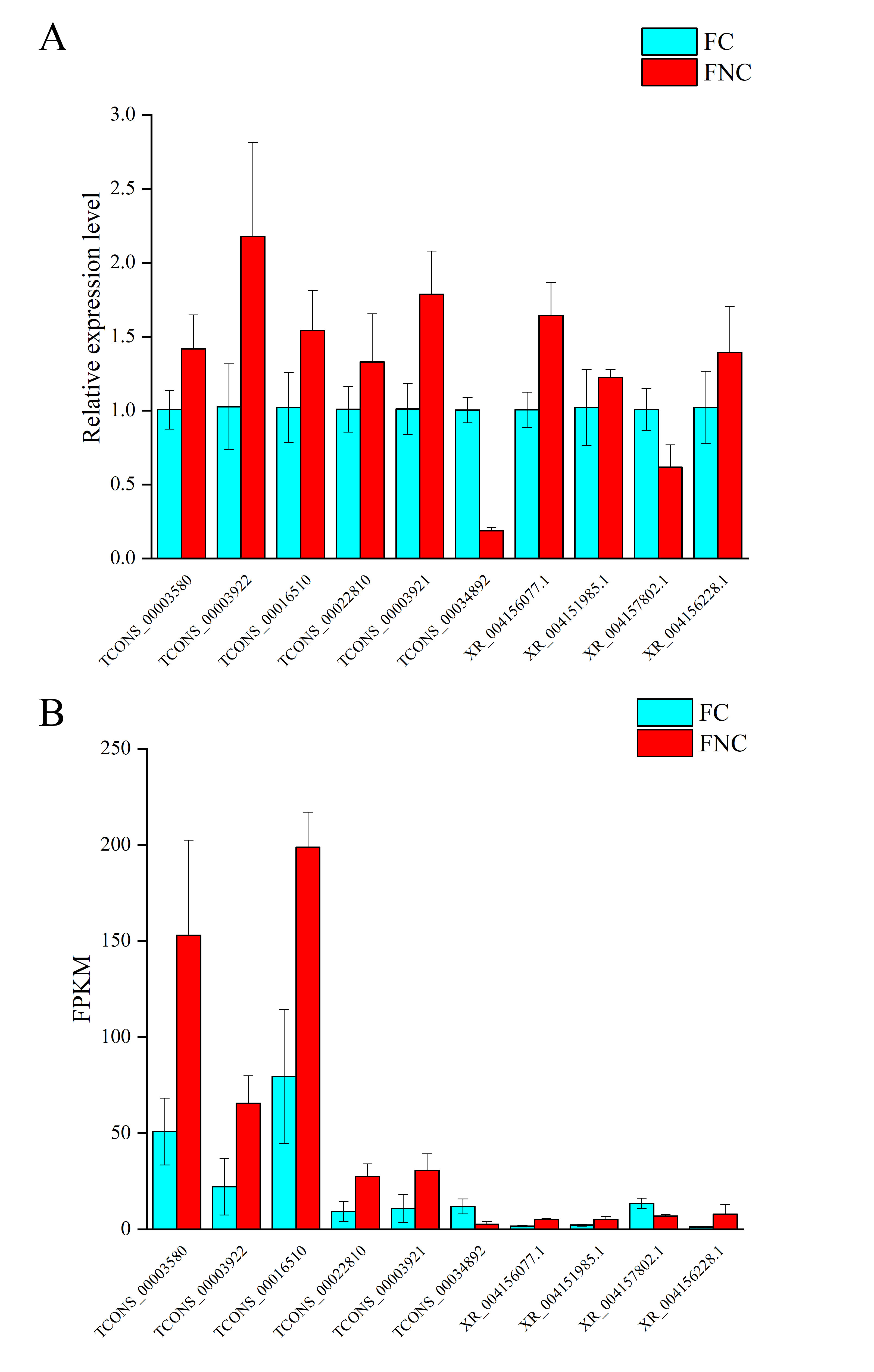

Supplement: Supplementary Figure 1 — The expression patterns of ten lncRNAs. [file DataSheet_1.zip › supplementary materials/Figure S1. The expression patterns of ten lncRNAs..jpg]
